# Unbiased Virus Detection in a Danish Zoo Using a Portable Metagenomic Sequencing System

**DOI:** 10.3390/v15061399

**Published:** 2023-06-20

**Authors:** Anna S. Fomsgaard, Stamatios A. Tahas, Katja Spiess, Charlotta Polacek, Jannik Fonager, Graham J. Belsham

**Affiliations:** 1 Department of Virus & Microbiological Special Diagnostics, Statens Serum Institut, 5 Artillerivej, 2300 Copenhagen, Denmark; 2 Department of Veterinary and Animal Sciences, University of Copenhagen, 4 Stigboejlen, 1870 Frederiksberg, Denmark; 3 Copenhagen Zoo, Roskildevej 38, 2000 Frederiksberg, Denmark

**Keywords:** point-of-care test (POCT), human–animal interface, field detection, cross-species transmission, nanopore sequencing, metagenomic sequencing

## Abstract

Metagenomic next-generation sequencing (mNGS) is receiving increased attention for the detection of new viruses and infections occurring at the human–animal interface. The ability to actively transport and relocate this technology enables in situ virus identification, which could reduce response time and enhance disease management. In a previous study, we developed a straightforward mNGS procedure that greatly enhances the detection of RNA and DNA viruses in human clinical samples. In this study, we improved the mNGS protocol with transportable battery-driven equipment for the portable, non-targeted detection of RNA and DNA viruses in animals from a large zoological facility, to simulate a field setting for point-of-incidence virus detection. From the resulting metagenomic data, we detected 13 vertebrate viruses from four major virus groups: (+)ssRNA, (+)ssRNA-RT, dsDNA and (+)ssDNA, including avian leukosis virus in domestic chickens (*Gallus gallus*), enzootic nasal tumour virus in goats (*Capra hircus*) and several small, circular, Rep-encoding, ssDNA (CRESS DNA) viruses in several mammal species. More significantly, we demonstrate that the mNGS method is able to detect potentially lethal animal viruses, such as elephant endotheliotropic herpesvirus in Asian elephants (*Elephas maximus*) and the newly described human-associated gemykibivirus 2, a human-to-animal cross-species virus, in a Linnaeus two-toed sloth (*Choloepus didactylus*) and its enclosure, for the first time.

## 1. Introduction

Emerging and re-emerging viral pathogens are being observed at an increasing rate. These pathogens can result in the spread of disease to both humans and other animals, as was the case with the zoonotic COVID-19 pandemic [1]. Furthermore, with ongoing changes in land-use, animal husbandry, traditional food markets, wildlife trade and the increased frequency of international travel, people and domestic animals are coming into contact with wildlife at a greater rate, which increases the likelihood of cross-species spillovers [2]. Activities at the human–animal interface, such as wildlife ecotourism, pet ownership and zoological gardens, present further risks of novel infections and new transmission routes [3,4,5,6,7]. The realization of interdependence between human and animal health is a key message in the One Heath approach. It is recognized that to sustain good human health, animal health must also be overseen, and vice versa. To achieve this, the One Health concept establishes important interdisciplinary collaborations across human, animal, environmental and governmental experts and stakeholders. These coordinated efforts will decrease the consequences of spillover events as they arise around the human–animal interface. The first line of defence is the early detection of the disease causative agent. An emerging virus may be detectable in the environment or in animals prior to being detected in humans, stressing the importance of animals as reservoirs for viruses that might cause zoonoses. Thus, increased surveillance and discovery of viruses, present in both healthy and diseased animals, is essential to sustain both animal and human health.

Metagenomic next-generation sequencing (mNGS) is a powerful technology for identifying new or unexpected pathogens [8]. An important augmentation is to make mNGS portable, which has been made possible with the Oxford Nanopore Technology (Oxford, UK) using the compact MinION long-read sequencing device [9]. Detecting viruses at the point of sampling allows for rapid identification and intervention as demonstrated during the 2014 Ebola outbreak in West Africa [10,11]. Additionally, field detection reduces potential virus degradation as well as risks associated with sample transportation. The proximity of the analysis further facilitates swift local actions, such as quarantines and contact tracing.

Zoological facilities most commonly exhibit multiple species of animals from more than one continent or biome. Daily husbandry, veterinary procedures, and interactions with the public or staff inevitably impose direct and/or indirect contact between species of animals that would never occur in their natural habitat. Several zoos have recently reported cases of SARS-CoV-2 in carnivores, artiodactyla and non-human primates that most likely occurred by transmission from humans [12]. In addition, synanthropic vertebrate species, such as the red fox (*Vulpes vulpes*), rodents and urban avian populations, are naturally drawn to zoos due to food availability and shelter. This has also created transmission routes as seen with, e.g., poxviruses, avian influenza and herpes virus transmission, from urban wildlife to rare zoo-housed exotic animal species that has, in some cases, been fatal and detrimental to species conservation [4,13,14,15,16,17,18]. The mNGS can provide a fast, point-of-incidence, surveillance system for biodiversity mixing pools, such as a zoo. Furthermore, the use of mNGS can provide a unique insight into the diversity of viruses co-existing in multiple animal species.

We previously developed a metagenomic microarray method for the unbiased detection of all known virus genomes described from animals and man [19]. However, the array technology requires continuous updating with new probes as viruses/variants are identified or mutate and the procedure is both time-consuming and labour-intensive. Previously, we developed a straightforward mNGS procedure that greatly enhances the detection of RNA and DNA viruses in clinical samples [20]. In the current study, we optimize the mNGS protocol using a transportable battery-driven equipment for the non-targeted detection of RNA and DNA viruses in animals from Copenhagen Zoo in Denmark, which exemplifies a field setting for point-of-incidence virus detection.

## 2. Materials and Methods

### 2.1. Collection and Processing of the Samples

Samples were collected from 37 different species in Copenhagen Zoo between September and November 2022 (Table 1). Samples from enclosures where zoo-housed animals were in close proximity to guests and/or urban wildlife as well as samples that could be obtained during veterinary interventions were prioritised. Sterile cotton swabs were used to obtain samples from oral, nasal or rectal/cloacal sites from individual animals opportunistically during veterinary interventions or where trained animal behaviour allowed. To screen as many individuals and areas as possible with the mNGS protocol, swabs taken simultaneously from individuals of the same species were processed in sub-pools. Fresh faecal samples were collected from an individual’s enclosure. Enclosure swabs were taken from fresh dung found in the morning in a stable. These enclosure swabs were pooled to represent the collection of a given species and, therefore, did not necessarily correspond to the number of individuals in the group but to the amount of fresh dung that had been produced at the time of sampling. Serum, full blood and tissue samples were obtained from animals during post-mortem examinations or, alternatively, from the Zoo’s archive in a frozen state. 

All fresh samples were processed on the day of collection. Swabs and biopsies were submerged within cryotubes containing PBS (700 µL) and shaken for 2 min. Aliquots were stored at −80 °C, or mixed 1:1 with MagNA Pure lysis binding buffer (MPLB-buffer, Roche Life Sciences). The guanidine iso-thiocyanate in the MPLB-buffer effectively inactivates viruses and proteins, including enzymes, and preserves RNA and DNA [21]. The remaining material was processed using the mobile mNGS virus detection method as described previously by Fomsgaard et al. [20] (described in Section 2.3, Section 2.4, Section 2.5 and Section 2.6) with some alterations to promote field deployability (see Section 2.4, Section 2.5 and Section 2.6; see Figure S1).

### 2.2. Microarray for the Validation of mNGS Findings

All samples found positive by mNGS for any vertebrate viruses were also analysed with an in-house panvirus microarray assay for supporting findings [19,22]. The panvirus microarray includes probes for the detection of >3000 complete genomes from vertebrate and invertebrate viruses obtained from GenBank (updated 2018). In addition, 40 out of 58 randomly selected samples that tested negative for vertebrate viruses by mNGS were also tested on the microarray.

At the time of collection, samples were mixed, 1:1, with MPLB-buffer and processed for analysis. Briefly, nucleic acids from the samples were extracted and purified on a MagNA Pure 96 instrument (Roche, Switzerland) and eluted in a 50 µL elution buffer from the MagNA Pure 96 DNA and Viral NA Small Volume Kit (Roche, Switzerland). Samples (20 µL) were used for separate random whole-genome transcriptome amplification (WTA) and whole-genome amplification (WGA) according to the REPLI-g Cell WGA & WTA kit protocol (Qiagen, Hilden, Germany). WTA- and WGA-amplified cDNA and DNA, respectively, were purified with the QiaAmp DNA mini-kit (Qiagen, Hilden, Germany) and 1.5 µg cDNA/DNA was labelled with either Cy3 or Cy5 using the SureTag DNA labelling kit (Agilent Technologies, CA, USA). Labelled samples were hybridized to the panvirus microarray (SurePrint G3 Custom Gene Expression Microarray (4 × 180 K), Agilent Technologies, Santa Clara, CA, USA), and the microarray data were processed as described previously [19,22].

### 2.3. Sample Pretreatment and Nucleic Acid Extraction

The samples were pretreated according to the field-deployable method described by Fomsgaard et al. [20] using an isothermal DNase I (Zymo Research, CA, USA) treatment for 15 min and 0.22 μM syringe filtration directly into the MPLB buffer. The nucleic acid (NA) was hand-extracted with the MagNA Pure LC Total Nucleic Acid Isolation Kit (Roche Life Sciences) as described by Rosenstierne et al. [23], which took approximately 10 min per sample. 

### 2.4. Whole-Transcriptome and Whole-Genome Amplification for RNA and DNA Viruses

Random amplification was performed with the REPLI-g Cell WGA & WTA Kit (Qiagen, Hilden, Germany), according to the manufacturer’s instructions but with the following alterations: (1) magnetic glass particles (MGPs) were kept in the input material [23], (2) the REPLI-g Cell WGA & WTA Kit’s cell lysis step was omitted and (3) 5′-phosphorylated random hexamers (pN6) (20 µM) were used instead of the oligo dT primers provided in the kit [19]. To make the mNGS procedure more portable, we used a miniPCR™mini8 samples thermal cycler (miniPCR bio™, Cambridge, MA, USA) with Sandberg All-in-1 Laptop Powerbank 24,000 power banks (Sandberg A/S, Birkerod, Denmark) for the temperature-dependent incubations. Each sample was processed in a parallel workflow for whole-transcriptome amplification (WTA) and whole-genome amplification (WGA) for RNA and DNA viruses, respectively. The WTA (cDNA) and WGA (DNA) products from each sample were mixed 1:1 after amplification to allow for the simultaneous detection of any RNA and or DNA virus present in a given sample during sequencing. The WTA and the WGA workflows were performed on separate miniPCR cyclers, allowing seven samples and a negative control to be processed in parallel. The REPLI-g incubation protocol was followed except that the ligation step was performed at 25 °C instead of 24 °C due to the miniPCR’s temperature range limit. The full REPLI-g protocol uses isothermal incubations with temperature ranges from 24 °C to 95 °C and takes 3 h and 50 min; this used approx. 50% of the power bank’s capacity.

### 2.5. Metagenomic Sequencing

Oxford Nanopore technology was used for fast and portable sequencing. Up to seven samples were multiplexed using the Rapid Barcoding Kit (SQK-RBK004; Oxford Nanopore Technology, Oxford, UK) without any bead-purification steps for protocol simplicity and a fast library prep of 10 min. Using a Qubit Fluorometer (Life Technologies, Cambridge, MA, USA), total DNA concentration for each sample was normalised to contain a maximum 400 ng of DNA before barcoding, as recommended for library preparation. We used R9.4.1 flowcells on the MK1C device (Oxford Nanopore Technology, Oxford, UK) with default settings and fast base calling enabled. The Minion MK1C was used in combination with two power banks that allowed for 10 h real-time sequencing.

A nasal swab from a Linnaeus’s two-toed sloth (*Choloepus didactylus*) was subsequently sequenced using the Nextera XT DNA Library Prep Kit (No. 15031942) to evaluate the presence of human-associated gemykibivirus 2 that was detected with the mNGS method.

### 2.6. Data Analysis

The fastq data files were de-multiplexed using MinKNOW (version 21.02.1) and then quality-trimmed using BBduk (version 38.84) using Geneious Prime (version 2022.1.1, https://www.geneious.com, Biomatters Ltd., Auckland, New Zealand) with a minimum quality score of 7 at each read’s end, as previously recommended by Geneious Prime for metagenomic-generated Nanopore reads and with read lengths above 100 nt. To be able to characterize thousands of reads in a less time-consuming and computational labour-intensive manner, the trimmed reads were BLASTed against the curated offline vertebrate virus Virosaurus database (version 98, 2020_4.2) [24]. This truncated the BLAST characterization to a few minutes on a standard 8 GB RAM portable PC. Hits with a minimum length of 100 nt and above 90% pairwise identity were further investigated. BLAST hit reference genomes were downloaded and the quality trimmed reads were mapped to the references using Minimap2 for long-read alignment [25]. For the Illumina-sequenced sample, paired reads were quality filtered with Phred score 30 and mapped to the human-associated gemykibivirus 2 genome sequence to obtain a consensus sequence for a phylogenetic analysis (see below).

Alignments were manually inspected, and mappings with evenly distributed horizontal coverage of a reference genome were considered true hits for an identified virus. Mappings only consisting of stacked reads in a small region of a reference sequence were considered false hits. To address the incomplete diversity of viruses in Virosaurus, the contigs generated from each mapping were BLASTed using the NCBI nt/nr database and inspected manually for the match between contigs and the reference sequences in order to find possible viruses not present in the Virosaurus database. If viruses scored higher in Bit-score than the Virosaurus-detected virus, the references of these viruses were collected, and the quality trimmed reads were re-mapped to the new reference, and the coverage, depth and virus speciation were inspected to determine whether they improved.

For gemykibivirus-positive samples, a phylogenetic analysis was performed on consensus sequences derived from three samples from the same animal (see Section 3.1) with genomes of other members of the gemykibivirus genus obtained from NCBI. The sequences were aligned using MAFFT and sites containing at least one gap were masked using the mask alignment tool in Geneious Prime. Randomized accelerated maximum likelihood with the general time-reversible (GTR) model and a 1000 bootstrap branch support was used.

## 3. Results and Discussion

In total, 130 samples were collected (from 45 enclosure swabs, 18 faecal swabs, 49 individual animal swabs (nasal, oral, cloacal/rectal), 15 fluid samples and 3 tissue samples). Some same species and enclosure swabs were sub-pooled (Table 1). A total of 71 samples from zoo-housed animals were processed in situ to identify vertebrate viruses using the mNGS method. Thirteen different viruses (Table 2) from four out of seven major virus groups (see Table 3: Baltimore groups; (+)ssRNA, (+)ssRNA-RT, dsDNA and (+)ssDNA [26]) were detected. The majority of DNA viruses detected were small (genome ~2 kb) circular DNA viruses from faecal and nasal swabs.

### 3.1. Case: Human-Associated Gemykibivirus 2 Found in Sloth

**Figure 1:**
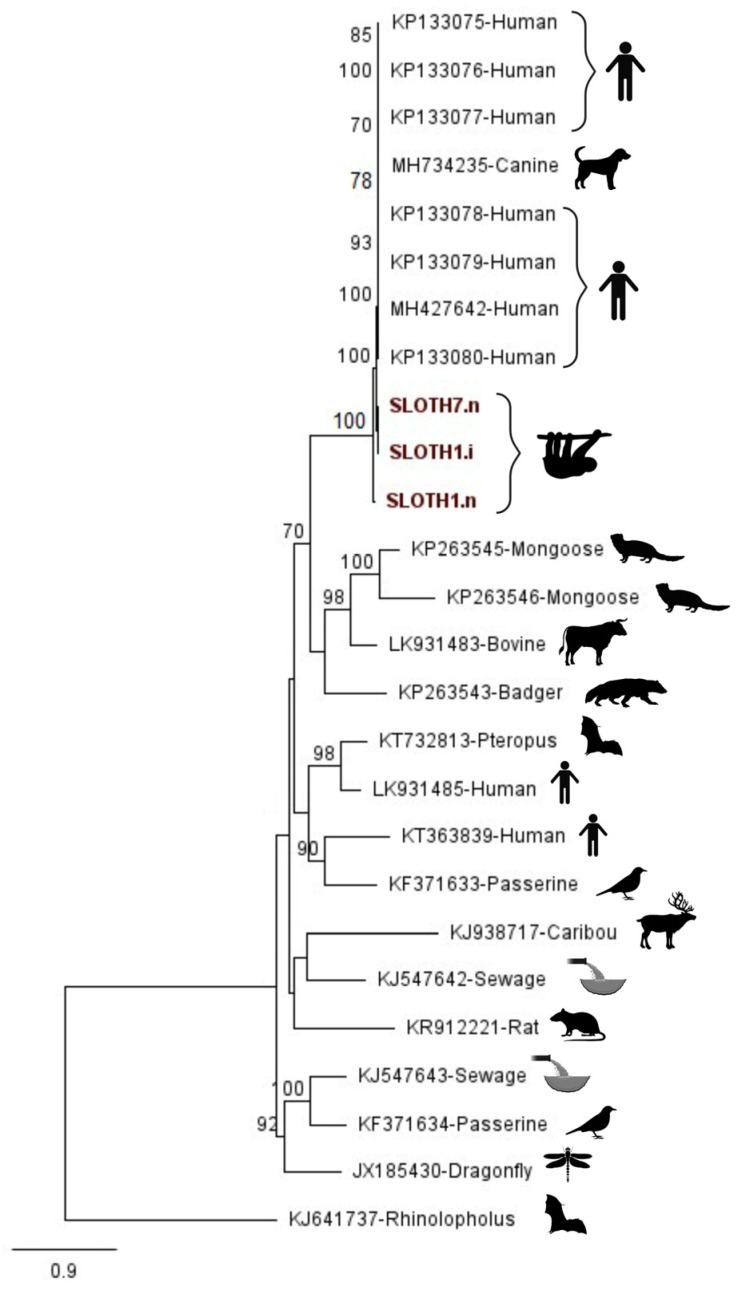
Phylogenetic relationships between gemykibivirus, detected in the present study from the described female Linnaeus’s two-toed sloth (*Choloepus didactylus*) and other members of the gemykibivirus genus. The numbers denote bootstrap values above 70 at a 1000 bootstrap branch support. The sequences generated in this study are marked in bold red font. Sloth1.i and Sloth1.n denote nasal samples from the sloth obtained with Illumina and nanopore sequencing, respectively. Sloth7.n denotes a nasal sample from the same individual collected four months later, post-mortem, generated with nanopore sequencing. Created with BioRender.com (accessed on 17 March 2023).

In September 2022, a female Linnaeus’s two-toed sloth (*Choloepus didactylus*) was sedated for the treatment of lower limb lacerations inflicted by a conspecific. A pooled oral and nasal swab was taken in which a human-associated gemykibivirus (GemyCV-SL1; Acc. No. MH734235) was detected. In total, 96.6% of the reference genome coverage was obtained (Table 2; sloth 1) using nanopore sequencing, compared to 100% coverage follow-up sequencing with Illumina (not shown). Three weeks later, the sloth was translocated from temporary hospitalisation facilities back to its enclosure. No virus was detected in a second nasal swab obtained during translocation. However, in January 2023, the sloth was found dead in its enclosure. An autopsy revealed no apparent infection and the cause of death was determined to be old age. The precise age of the sloth had never been determined; however, it was introduced to the zoo during the 1980s and its approximate age was estimated to surpass 38 years when it died. A nasal swab taken during post-mortem examination contained the virus again, with 84.3% genome coverage (Table 2; Sloth 7). There was no detection of GemyCV-SL1 in the mouth, liver, kidneys or rectum of the deceased sloth. Phylogenetic analysis (Figure 1) grouped the consensus sequences from the sloth’s nasal swabs with human isolates from a throat swab (Acc. No. MH427642), cerebral spinal fluid (Acc. No. KP133076-KP133077), diarrheal samples (Acc. No. KP133078-KP133079), sewage (Acc. No. KP133080) and a pool of serum from healthy pet dogs in Brazil (Acc. No. MH734253). GemyCV-SL1 has been detected in various human samples [27,28,29,30], including sterile sites such as cerebral spinal fluid, but the clinical significance of the virus infection is not established. To date, only one case study of a patient with respiratory distress provided evidence for the virus being the causative agent of disease [29]. The virus has also been detected in pooled serum samples from healthy companion dogs in Brazil (Acc. No. MH734235), providing the first evidence of this virus being able to infect different species [31].

An environmental sub-pool of swab samples was taken from the feeding stations in the enclosure and the microarray system revealed the presence of GemyCV-SL1. Although it cannot be determined whether the sloth had the virus in the nasal cavity as an environmental contamination or an infection, it could be considered that being away from the enclosure for three weeks enabled the sloth to clear the virus while the return to the enclosure might have resulted in re-infection. This was also supported by the results of the phylogenetic analysis (Figure 1), in which it was apparent that the same virus was found in the sloth at the different timepoints. However, it cannot be excluded that a low viral load or discontinuous viral shedding could be why the mNGS method and microarray system did not detect GemyCV-SL1 in the sloth at the hospitalisation facility. The sloth habitat is a walk-through enclosure open to the public and shared with two other, younger sloths and various avian and reptile species. It would be interesting to see whether the remaining sloths have this virus and whether it can also be found in the other species residing in the open enclosure. However, the sloths in the Zoo are seldom restrained; thus, the case of the female and any further update and sampling of other sloths would have to be opportunistically investigated.

### 3.2. Diverse Viruses Identified from Animals at the Copenhagen Zoo

#### 3.2.1. RNA Viruses

Viral contigs generated from enclosure swabs collected in the chicken (*Gallus gallus*) enclosure matched multiple metagenomic-assembled genomes (MAG) or partial coding regions of viruses from the family *Picornaviridae* (Table 2). These viruses have small, positive-sense, RNA genomes (ca. 7–9 kb). The comparison of the various MAG viruses with the Virosaurus database indicated the reads matched those of multiple *Picornaviridae* members, e.g., chicken megrivirus strain chicken/NLD/2019 (Acc. Nos. MW684798k, MW054506-MW054511), chicken picornavirus 4 isolate 5C (Acc. No. KF979335), Melegrivirus A (Acc. No. KF961188) and megrivirus C (or chicken picornavirus 5; Acc. No. MH806866), which is thought to be a naturally occurring recombinant picornavirus with parts of its genome derived from Melegrivirus A, but has been classified as a megrivirus [32]. These results are comparable with other mNGS studies that have detected multiple MAG from *Picornaviridae* in both healthy groups and groups with runting–stunting syndrome or enteritis [33,34]. The chicken coop was closed to the public while the chickens were free to roam around in the Zoo. There have not been any reports of pathogenic infections in humans by these viruses and their potential for being pathogenic in other avian species is still unclear.

#### 3.2.2. Retroviruses

Avian leukosis virus (ALV) was detected in the same enclosure swab samples from the chicken coop as described above, with reads assigned to ALV and the chicken (*Gallus gallus*) genome. The microarray system also detected a weak positive signal for other avian leukosis/sarcoma viruses (avian leukaemia virus retrovirus, Rous sarcoma retrovirus and Y73 sarcoma retrovirus), indicating either a coinfection or that distinction between these closely related viruses was unattainable. ALV has a positive-sense RNA genome (7.6 kb) and is naturally occurring, worldwide, in chickens and other Galliformes [35]. ALVs can induce non-specific clinical signs, such as lethargy, reduced egg production and lymphoid neoplasia in chickens, but these viruses are not known to spillover to other taxonomic classes.

The Enzootic nasal tumour virus (ENTV-2) of goats and the Jaagsiekte sheep retrovirus (JSRV) were detected with the mNGS method and confirmed with the microarray system in a sub-pool of nasal swabs from three goats. However, no contigs derived from either alignment of reads to ENTV-2 or JSRV reference genomes were assigned to JSRV in the NCBI nr/nt database. Both viruses are members of the *Retroviridae* family, with ssRNA-RT genomes (7.2–7.4 kb). The genomes are 88.8% identical; so, to investigate mis-assignment, the mNGS reads were remapped to JSRV and ENTV-2. The contigs derived from the read mapping to the JSRV reference genome were once more not assigned to JSRV but to ENTV-2. Individuals infected with ENTV-2 can shed the virus through nasal secretions, which was the material in which the virus was detected in this study. Detecting ENTV-2 in this study was not surprising, since it is naturally occurring in goats worldwide except in Oceania [36]. JSRV normally only infects sheep, except for a few cases [37,38], and the contigs for the mapping refinement suggest that reads apparently belonging to ENTV-2 were mis-assigned to JSRV. Both viruses can induce tumours that slowly develop and cause respiratory distress and deformations in the eyes and skull. The goats are kept in an open enclosure in the Children’s Zoo that visitors can enter. As these retroviruses are not zoonotic, it is not a public health concern, but it is useful to know the presence of the viruses to avoid the unintentional spread of the disease to previously unaffected flocks or new geographical regions [36,37,39].

#### 3.2.3. DNA Viruses

Elephant endotheliotropic herpesvirus (EEHV) 1 and 4 were detected in frozen samples from three juvenile male Asian elephants (*Elephas maximus*) that had died from haemorrhagic disease. Both the mNGS protocol and the microarray system detected EEHV-1 in two of these cases (Elephant 2 and 3), whereas EEHV-4 was only detected with the mNGS protocol. This is surprising, considering the high viral load in both the serum sample and pericardial fluid identified by an EEHV-specific qPCR (Table 4). The samples from Elephant1 were the oldest available for EEHV investigation. Although unclear, DNA fragmentation caused by multiple freeze–thaw cycles and/or prolonged storage may explain the inability of the microarray system to detect EEHV-4. Furthermore, for a virus with a large genome, such as EEHV-4 (>200,000 bp long), the gaps between the microarray-probes might be large enough for viral reads to map in between probes. In addition, the detection threshold for the microarray system requires multiple probe matches for a positive signal. It is possible that variation from the reference sequence will mean not all probes will efficiently bind to the sample sequence, thus reducing the positive signal of a few EEHV-4-binding probes below the microarray’s detection threshold. The EEHV is an ubiquitous virus of Asian elephants. When transmitted to young Asian elephants between the ages of 2 and 8, it can result in a haemorrhagic condition that can be fatal [40]. As many as 25 juvenile elephants died in captivity due to EEHV infection between 1983 and 2017 in Europe [41]. The early identification of high viremia, alongside other clinical and haematological indicators, are crucial for the initiation of early treatment, e.g., with antivirals and supportive IV fluids and increasing the chances of survival in afflicted juveniles. Thus, for these particular viruses, when immediate detection is of uttermost importance for the survival of an individual, a virus-specific real-time PCR or loop-mediated isothermal amplification (LAMP) test would be the current recommended choice. However, it is noteworthy that the mNGS method was able to detect the viruses as well as, or even better (for EEHV-4 in Elephant1; Table 2) than, the microarray system.

#### 3.2.4. CRESS DNA Viruses

CRESS (Circular, Small, Rep-Encoding, ssDNA) DNA viruses are a group of viruses that have been detected in a diverse group of hosts (vertebrates, invertebrates, plants and fungi) and in environmental samples. They are characterized by their small ssDNA genome size (range of 1–6 kb), fast substitution rates (a rate of 1.2 × 10^−3^ substitutions/site/year has been estimated for porcine circovirus) and a replication initiator protein [42,43,44]. This study identified five viruses from the CRESS DNA families *Circoviridae, Genomoviridae* and *Smacoviridae*. Since this study focused on vertebrate viruses, the presence of microalgae-infecting *Bacilladnaviridae* and plant-infecting *Geminiviridae* and *Nanoviridae* was not investigated.

*Circoviridae.* Horse-associated cyclovirus 1 (CyCV Equ1) was detected from the nasal swabs obtained from asymptomatic goats in the Children’s Zoo with complete genome coverage (Table 2). Despite the full genome coverage of CyCV Equ1, the microarray system did not detect this virus. A sequence assessment showed mismatches between the microarray probes and the CyCV Equ1, which could explain the lack of detection and be a consequence of the fast substitution rates of CRESS DNA viruses (compared to average dsDNA virus estimated at 10^−8^ substitutions/site/year [45]). Such a mutation rate may have caused probe mismatches that could be detrimental for the detection of the virus, especially when there are a smaller number of probes along a small genome such as the CyCV Equ1 as opposed to larger genome sizes that would have more probes to bind at non-mutated sites [45]. CyCV Equ1 was first detected in the USA in a sub-pool of horse nasal swabs material with respiratory problems and, when investigated further, in individual nasal secretion and faeces [46]. However, the study did not conclude whether the cyclovirus was the cause of the respiratory distress or merely an environmental contamination of the nasal secretion.

*Genomoviridae*. Four viruses of the *Genomoviridae* family were detected: sewage-associated gemycircularvirus-4, faeces-associated gemycircular virus 17, MAG: *Genomoviridae* sp. isolate ctba76 and human-associated gemykibivirus 2 (Table 2). Sewage-associated gemycircularvirus-4 (SaCM-4) was detected from goat nasal swab samples with full genome coverage (Table 2). This virus was first found in a sewage oxidation pond in New Zealand and the sequence predicted 53% amino acid identity in its Rep-protein with a plant virus (Acc. No. KC979000), which belongs to the *Nanoviridae* family [47]. SaCM-4 might be associated with a type of plant virus that had been ingested with food; it was found in the nasal cavity of herbivores, such as goats, but the origin or clinical significance of its presence was not conclusive.

Faeces-associated gemycircular virus 17 (FaGmV-17) was detected in a nasal swab from a single horse. The virus was not detected eight weeks later when the same horse was sampled again. It was first detected in chicken faecal swabs in New Zealand but its host specificity is not known and it has not been linked to any clinical disease [47]. The chickens in Copenhagen Zoo are housed next to the horse stables with an outdoor area where the chickens are free to roam. Finding the virus in a non-sterile location, such as the nostril of a single horse, which was absent eight weeks later might simply indicate a contact point between chicken faeces and the horse muzzle. FaGmV-17 was not detected in the chickens, but these samples were taken concurrent with the second horse nasal swabs that also lacked this virus.

The virus MAG: *Genomoviridae* sp. isolate ctba76 was detected in nasal swabs from three horses and one pig with full-genome coverage (Table 2). The virus was not detected using the microarray system since this virus was not represented on the array. This virus was not represented in the Virosaurus database either and initially the database returned BLAST hits to another gemycircularvirus represented in the database, Pteropus-associated gemycircularvirus 3 (Acc. No. NC_038486). However, with the refining BLAST of the generated contigs, it was clear that the contigs belonged to the MAG: *Genomoviridae* sp. isolate ctba76 (Acc. No. MK032755). Remapping the reads for each sample improved the reference coverage to 100% for all four samples. The virus was originally identified in environmental swabs in and around wild mice in the USA [48]. As rodents are free-living urban wildlife, especially in a zoo where hay and food are readily available, the presence of this virus in animals that consume and live in the presence of hay can be expected. MAG: *Genomoviridae* sp. isolate ctba76 is not associated with any known disease and is therefore not a concern currently, but its presence in several different mammals could indicate that this virus might be more ubiquitous than previously considered.

The microarray system detected GemyCV-SL1 in the sloth (Section 3.1), while the other members of *Genomoviridae* could not be detected due to the absence of probes for these viruses.

*Smacoviridae.* Viral contigs derived from a fresh faecal sample from a juvenile male Lar gibbon (*Hylobates lar*) showed high similarity with Gorilla-associated porprismacovirus 1 with 52.6% horizontal reference coverage. It was first described from a gorilla stool sample in San Francisco Zoo alongside other CRESS DNA viruses in other non-human primates [49]. It is unclear whether the smacoviruses infect the host organism or simply originate from contaminated food sources [49]. However, one study showed that smacoviruses were detected in cattle stool samples but not in their food [50], and another study showed that only a subset of pigs fed the same feed had smacovirus in their stools [51] arguing against contaminated food origin and suggesting that they originated from enteric cells. A mouth swab sample from the gibbon was also collected concurrently, but no virus was detected in this. The significance of the detection of a smacovirus in faecal samples from two non-human primate species is unknown, but it is evident that they are not uncommon. The microarray system did not detect the Gorilla-associated porprismacovirus 1 due to the absence of probes.

**Table 3:** Detected vertebrate virus descriptions with a common name, their family and Baltimore group that classify viruses according to their genome with positive (+) or negative (−) strand orientation and whether the genome is single-stranded (ss) or double-stranded (ds) genome composed of RNA or DNA and replicate via reverse transcription (RT). The first recording of host species for each virus and its literature reference are provided in separate columns.

Virus Detected in This Study	Family	Baltimore Group	Genome Structure	First Reported Host Species	Reference
Avian leukosis virus	*Retroviridae*	ssRNA-RT	Non-segmented	*Gallus gallus*	[52]
Elephant endotheliotropic herpesvirus 1	*Herpesviridae*	dsDNA	Non-segmented	*Elephantidae*	[53]
Elephant endotheliotropic herpesvirus 4	*Herpesviridae*	dsDNA	Non-segmented	*Elephantidae*	[53]
Enzootic nasal tumour virus of goats	*Retroviridae*	ssRNA-RT	Non-segmented	*Capra hircus*	[54,55,56]
Faeces-associated gemycircularvirus 17	*Genomoviridae*	ssDNA	Circular, Non-segmented	*Gallus gallus*	[57]
Gorilla-associated porprismacovirus 1	*Smacoviridae*	ssDNA	Circular, Non-segmented	*Gorilla gorilla*	[49]
Horse-associated cyclovirus 1	*Circoviridae*	ssDNA	Circular, Non-segmented	*Equus caballus*	[46]
Human-associated gemykibivirus 2	*Genomoviridae*	ssDNA	Circular, Non-segmented	*Homo sapiens*	[28]
Jaagsiekte sheep retrovirus	*Retroviridae*	ssRNA-RT	Non-segmented	*Ovis aries*	[58]
Picornavirus 4	*Picornaviridae*	ssRNA(+)	Non-segmented	*Gallus gallus*	[34,59]
Picornavirus 5/Megrivirus	*Picornaviridae*	ssRNA(+)	Non-segmented	*Gallus gallus*	[32]
MAG: *Genomoviridae* sp. isolate ctba76	*Genomoviridae*	ssDNA	Circular, Non-segmented	Wild mouse	[Accession No. MK032755]
Sewage-derived gemycircularvirus 4	*Genomoviridae*	ssDNA	Circular, Non-segmented	Acquired from environmental sample(s)	[47]

**Table 4:** CT-values from Copenhagen Zoo’s elephant endotheliotropic herpesvirus (EEHV) real-time qPCR assays performed after the thawing of archived samples in 2022.

	Year of Death	Sample Material	EEHV-1	EEHV-4	EEHV-5
Elephant 1	2003	Serum	30.5	28.8	33.3
		Pericardial fluid	30.7	28.8	33.3
Elephant 2	2014	Serum	21.2	No CT	32.8
Elephant 3	2022 (August)	Heart	19.2	No CT	No CT

### 3.3. Considerations and Future Perspectives

The mNGS field method and the microarray system both use the REPLI-g with the phi29 enzyme for random nucleotide amplification as do other virus metagenomics studies [60,61]. The enzyme’s multiple displacement amplification may favour the detection of small circular DNA viruses. In fact, six circular DNA viruses were identified in this study. However, several RNA viruses as well as linear dsDNA viruses were also detected (Table 2) and the mNGS method’s ability to detect the SARS-CoV-2 RNA and human papillomavirus (HPV) DNA in diagnostic clinical samples has been previously demonstrated [20]. This shows the versatility of this mNGS approach to detect a wide range of different viruses. Alternatively, such circular DNA virus could be very abundant as suggested by other metagenomic virus studies of environmental samples [47,57]. As more metagenomic virus screenings are made, the abundance of CRESS viruses should be outlined more precisely.

The microarray system detected 7 of the 13 viruses identified by the mNGS protocol, while 6 viruses were not identified. In four cases, this was potentially due to degraded frozen samples, but also decreased probe specificity or absent probes. However, the microarray system did detect multiple different group A rotaviruses from different host species in three samples (oral swabs, rectal swabs and serum) from a sub-pool of four Egyptian fruit bats (*Rousettus aegyptiacus*), which the mNGS method did not detect. Subsequent real-time qPCR assays for human rotavirus A produced negative results for oral swabs (no CT-value) but confirmed the presence of rotavirus A in rectal swabs (CT = 31.7) and serum (CT = 31.9). Although the human rotavirus A qPCR could confirm some of the microarray findings, this qPCR may not be optimal for the quantification of bat rotaviruses. Both the mNGS method and the microarray system were developed for the detection of viruses in individuals with clinical symptoms, with the assumption that the viral load would be higher during disease and thus more readily detected [20]. The relatively high CT-values obtained in the rotavirus A qPCR indicate a viral load that is too low to be detected using the mNGS protocol, which targets free virions in comparison to targeted diagnostic assays where usually the intracellular viral NA is also included [20].

The use of MPLB buffer for hand-held NA extraction was initially developed for the targeted detection of Ebola virus RNA in whole blood and urine samples [23]. In our previous work, where we developed the mNGS protocol for virus detection [20], we showed the use of the field extraction method on oropharyngeal, vesicular and cervical swab materials for the downstream processing and detection of SARS-CoV-2 RNA and HPV DNA with and without coinfection with molluscum contagiosum virus. That study suggested investigations of the protocol’s ability to include other sample materials. In this paper, we expanded the mobility and flexibility of the protocol by the incorporation of power banks and the miniPCR equipment, and further showed the ability of the mNGS protocol to detect DNA and RNA viruses in tissue samples, faecal samples and nasal swabs from a variety of animal species.

During this two-month study, we did not aim to sample all 230 different animal species in the Copenhagen Zoo [62]. The collection of non-invasive faecal samples from animals in outdoor enclosures, where humans and urban wildlife were in close contact, was prioritized. We also screened all animals that visited the zoo’s veterinary clinic even when no apparent symptoms of infection were observed since asymptomatic virus infections can occur, e.g., human cytomegalovirus infection in about 60% of the Danish population [63] or SARS-CoV-2 [64]. This study provided a baseline for what can be detected in a zoo utilizing a portable mNGS system with a fast response time. The mNGS workflow presented in this paper includes 15 min for pretreatment, 10 min for NA extraction per sample, 4 h for whole-genome and whole-transcriptome amplification and 10 min for library preparation. With setup and hand-on steps, it takes about 7 h to perform in comparison to the microarray system, which takes 3.5 days, with reasonable working hours, after the sample is delivered to the laboratory. Regular screening and pathogen surveillance at a human–animal interface, as in zoos, enables advances in animal welfare and public health. A consideration when using mNGS for virus detection is the recognition that the number of viral reads is proportional to the viral load in combination with the virus genome size and the proportion of non-viral reads that are generated. This is apparent in Table 2, in which the viruses with smaller genomes have higher coverage and depth than viruses with larger genomes. High coverage and depth can be important if, e.g., typing and variant calling are needed. However, a few good-quality reads at species-specific areas can be sufficient for virus detection. The use of mNGS is useful for precisely this. If confirmation or further whole-genome characterization is needed, an aliquot of the sample in MPLB buffer can be stored and then processed at more specialized facilities while early stage management guidelines are being implemented. Conveniently, room-temperature storage in the MPLB buffer is recommended [21].

For the further application of this mNGS field protocol, we suggest the use of this protocol in geographical regions where advanced diagnostic laboratory infrastructure is lacking and where biodiversity hotspots with an increased risk of viral pathogen spillovers are present.

## 4. Conclusions

With an optimized mNGS method for fast virus detection using a portable system, this study set out to investigate which viruses were present or circulating in Copenhagen Zoo as a test-of-concept. Thirteen different vertebrate viruses, representing four Baltimore classification groups, were detected. While the presence of virus commonly occurring in host species, such as AVL in chickens and ENTV-2 in goats, was expected, the presence of viruses, such as CRESS DNA viruses, were interesting and their abundance could indicate that these viruses are part of our normal environment and hence also in a zoological facility. It was, furthermore, shown that the mNGS method was readily able to detect potentially lethal animal viruses, such as EEHV in Asian elephants as well as the newly described GemyCV-SL1, a human-to-animal cross-species virus, in a Linnaeus two-toed sloth for the first time. It is envisaged that this portable mNGS system will allow for rapid on-site virus identification and thus may help to mitigate large outbreaks.

## Figures and Tables

**Table 1:** Sample collection details for the screening of viruses in Copenhagen Zoo in 2022. Samples: number of samples processed with the mNGS method. Sub-pools: number of pooled samples per processed sample. Population: number of individuals of the same species in the enclosure. NA (not applicable) population size for older samples obtained from the Zoo’s archive in a frozen state. (*) frozen samples.

Sample Type	Material	Species	Samples	Sub-Pool	Population
Swabs	Enclosure	American flamingo (*Phoenicopterus ruber*)	1	8	80
		Black-capped squirrel monkey (*Saimiri boliviensis*)	1	4	12
		Blesbok (*Damaliscus pygargus phillipsi*)	1	1	2
		Chicken (*Gallus gallus domesticus*)	1	5	13
		Grant’s zebra (*Equus quagga boehmi*)	1	2	3
		Humboldt penguin (*Spheniscus humboldti*)	1	8	40
		Kea (*Nestor notabilis*)	1	4	3
		Llama (*Lama glama*)	1	2	2
		Ostrich (*Struthio camelus camelus*)	2	1	2
		Reticulated giraffe (*Giraffa camelopardalis reticulata*)	1	4	5
		Sable antilope (*Hippotragus niger*)	1	3	5
		Linnaeus’s two-toed sloth (*Choloepus didactylus*)	1	1	3
		Southern white rhinoceros (*Ceratotherium simum simum*)	1	1	4
	Faecal	Amur Leopard (*Panthera pardus orientalis*)	1	1	3
		Amur tiger (*Panthera tigris altaica*)	1	1	2
		Caracal (*Caracal caracal*)	2	1	2
		Eurasian brown bear (*Ursus arctos arctos*)	1	1	3
		Golden marmoset (*Leontopithecus rosalia*)	1	1	2
		Linnaeus’s two-toed sloth (*Choloepus didactylus*)	1	1	4
		Hamaydrian baboon (*Papio hamadryas*)	1	3	17
		Lion (*Panthera leo*)	1	1	4
		Muskox (*Ovibos moschatus*)	1	1	3
		Polar bear (*Ursus maritimus*)	1	1	4
		Rock wallaby (*Petrogale xanthopus*)	1	1	4
		Giant panda (*Ailuropoda melanoleuca*)	2	1	2
		Gibbon (*Hylobates lar*)	1	1	5
		Hippopotamus (*Hippopotamus amphibius*)	1	1	5
	Nasal	Linnaeus’s two-toed sloth (*Choloepus didactylus*)	2	1	3
		Hippopotamus (*Hippopotamus amphibius*)	1	1	5
		Goat (*Capra hircus*)	1	3	26
		Horse (*Equus ferus caballus*)	4	1	5
		Pig (*Sus scrofa domesticus*)	1	1	1
		Southern white rhinoceros (*Ceratotherium simum simum*)	2	1	4
	Oral	Chimpanzee (*Pan troglodytes*)	1	1	11
		Giant panda (*Ailuropoda melanoleuca*)	2	1	2
		Linnaeus’s two-toed sloth (*Choloepus didactylus*)	2	1	3
		Hippopotamus (*Hippopotamus amphibius*)	1	1	3
		Egyptian fruit bat (*Rousettus aegyptiacus*)	2	4	150
	Nasal/oral	Linnaeus’s two-toed sloth (*Choloepus didactylus*)	2	1	3
		Gibbon (*Hylobates lar*)	1	1	5
	Cloacal	Crested Pigeon (*Ocyphaps lophotes*)	2	3	6
		Galah parrot (*Eolophus roseicapilla*)	1	2	2
		Tasmanian devil (*Sarcophilus harrisii*)	1	1	5
	Rectal	Egyptian fruit bat (*Rousettus aegyptiacus*)	2	4	150
		Linnaeus’s two-toed sloth (*Choloepus didactylus*)	1	1	3
		Vari (*Varecia variegata*)	1	1	7
Fluids	Urine	Egyptian fruit bat (*Rousettus aegyptiacus*)	1	2	150
	Full blood *	Ball python (*Python regius*)	2	1	NA
	Serum	Egyptian fruit bat (*Rousettus aegyptiacus*)	2	4	150
	Serum *	Asian Elephant (*Elephas maximus*)	2	1	NA
	Pericardial fluid *	Asian Elephant (*Elephas maximus*)	1	1	NA
Tissue	Heart *	Asian Elephant (*Elephas maximus*)	1	1	NA
	Kidney	Linnaeus’s two-toed sloth (*Choloepus didactylus*)	1	1	NA
	Liver	Linnaeus’s two-toed sloth (*Choloepus didactylus*)	1	1	3

**Table 2:** Detection of vertebrate viruses in samples from different animals at Copenhagen Zoo with the mNGS workflow. Virus accession number denotes the reference genome that the reads from each sample were mapped to after Virosaurus BLAST detection. Shown is the reference genome coverage by reads and the mean depth of coverage across the entire reference genome. Total reads are the number of quality-trimmed reads generated for each sample. The pairwise identity percentage range is shown between assembled contig(s) and virus reference obtained from NCBI nr/nt. NA denotes the absence of probes for these viruses.

Sample	Sample Material	Virus Species Detected	Virus Accession Number	Reference Genome Size	Reference Genome Coverage (%)	Mean Depth	Viral Reads Mapped	Total Reads	Pairwise Identity% Range between Contigs and NCBI	Microarray Confirmed
Elephant 1	Serum	Elephant endotheliotropic herpesvirus 4	KT832477	205,896 bp	8.9	0.4	557	87,808	86.7–100.0	No
	Pericardial fluid				0.6	0.1	46	112,835	90.3–100.0	No
Elephant 2	Serum	Elephant endotheliotropic herpesvirus 1	KC462165	180,421 bp	0.3	0.0	25	89,629	95.4–99.1	Yes
Elephant 3	Heart				3.6	0.3	212	58,492	86.6–100.0	Yes
Chicken	Enclosure swab	Avian leukosis virus	KU937324	7669 nt	2.8	0.0	2	107,628	96.0–98.3	Yes
		Picornavirus 4	KF979335	9564 nt	26.9	0.9	38	107,666	89.6–91.7	Yes
		Picornavirus 5/megrivirus	MH806866	9567 nt	10.5	0.5	25	107,656	90.5–94.4	Yes
Gibbon	Faecal swab	Gorilla associated porprismacovirus 1	KP233191	2532 bp	53.4	2.6	26	61,534	90.3	NA
Goat	Nasal swab	Enzootic nasal tumour virus of goats	MK164400	7279 nt	27.7	4.2	92	95,465	86.3–98.1	Yes
		Jaagsiekte sheep like-retrovirus	DQ838494	7430 nt	48.4	38.5	536	95,710	90.4	Yes
		Horse associated cyclovirus 1	KR902499	1843 bp	100	13.4	37	95,419	97.8	No
		Sewage derived gemycircularvirus 4	KJ547634	2115 bp	100	12.1	103	95,463	96.8	NA
Horse 1	Nasal swab	Faeces associated gemycircularvirus 17	KT862242	2230 bp	100	18.0	76	68,170	92.2	NA
		MAG: *Genomoviridae* sp. isolate ctba76	MK032755	2176 bp	100	27.7	133	68,170	92.1	NA
Horse 2	Nasal swab				100	42.7	1959	92,294	91.5	
Horse 3	Nasal swab				100	60.8	3047	112,632	92.3	
Pig	Nasal swab				100	71.9	539	142,068	97.2	
Sloth 1	Nasal swab	Human associated gemykibivirus 2	MH734235	2210 bp	96.6	11.3	88	23,782	91.3–96.0	Yes
Sloth 7	Nasal swab				84.3	16.4	133	177,374	98.9	Yes

## Data Availability

Data can be made available upon reasonable request to the corresponding authors.

## Supplementary Materials

The following supporting information can be downloaded at: https://www.mdpi.com/article/10.3390/v15061399/s1, Figure S1: Equipment and reagents in the portable mNGS workflow.
