# Supplementary material for: Unbiased Virus Detection in a Danish Zoo Using a Portable Metagenomic Sequencing System
Source: Viruses. 2023 Jun 20;15(6):1399. doi: 10.3390/v15061399 (PMC10303153; doi:10.3390/v15061399)
Supplement: Supplementary file 1 [file viruses-15-01399-s001.zip › viruses-2421486-supplementary.pdf]

Supplementary Figure S1.

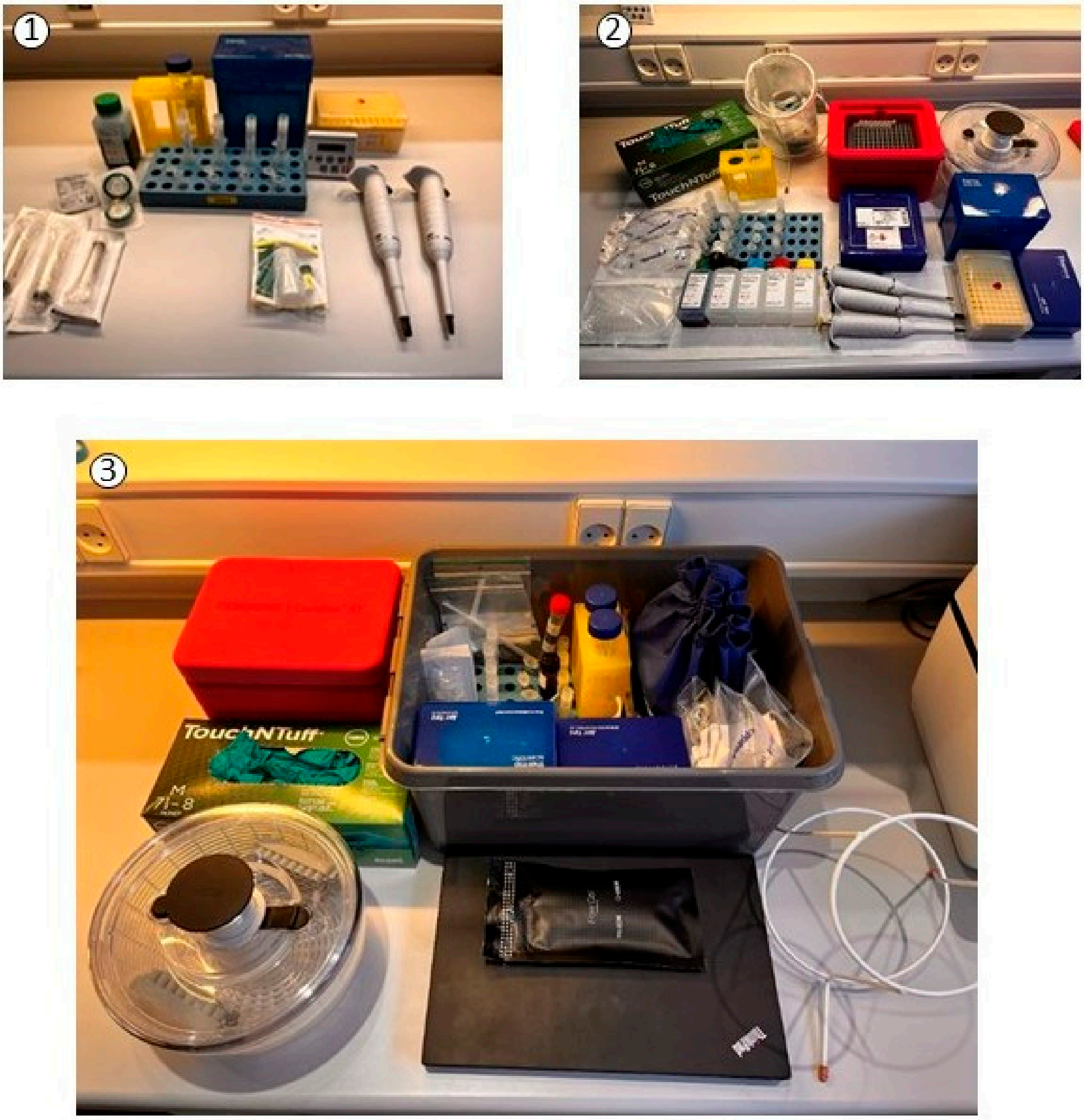

The equipment and reagents used for the mNGS workflow are presented unpacked in pictures 1) pretreatment necessities and 2) necessities for NA extraction and REPLI-g amplification. In picture 3 the items shown in 1) and 2) are packed to be transported with REPLI-g reagents and nanopore barcodes in the cooling box (red). The grey transportation box in Picture 3 contains

- Sterile cotton swabs.
- Cryotubes to hold used cotton swabs.
- A bag with 1.5 ml Eppendorf tubes for various uses.
- A bag with 0.2 ml PCR tubes with lids for various uses.
- One 10ul, one 100 ul and one 1000 ul pipette
- 10ul, 100 ul and 1000 ul pipette tips
- Trash bags for used pipette tips

- An Eppendorf tube holder for working with samples collected, and transporting NA extraction reagents
- A 50 ml Nunc tube holder with one Nunc tube with PBS and one empty Nunc tube for collecting NA extraction waste.
- Reagents and pretreatment-specific equipment; DNase I set, 5 ml syringes, filters and a clock.
- NA extraction reagents aliquoted in cryotubes or Eppendorf tubes.
- A miniPCR® mini8 thermal cycler in a blue transport bag
- Three Sandberg All-In-1 Laptop Powerbank 24000 power banks to power a miniPCR and an MK1C MinION and a spare

The remaining items outside the grey transportation box and the red cooling box are

- Disposable gloves
- A salad swing used as a hand-driven centrifuge
- A portable 8 GB RAM PC
- An Oxford Nanopore flowcell
- A trash bag holder

Not shown in the picture is the Oxford Nanopore Technology MinION Mk1C used for sequencing.
